# All-Optical Frequency Modulated High Pressure MEMS Sensor for Remote and Distributed Sensing

**DOI:** 10.3390/s111110615

**Published:** 2011-11-08

**Authors:** Kasper Reck, Erik V. Thomsen, Ole Hansen

**Affiliations:** 1 DTU Nanotech, Technical University of Denmark, Øersteds Plads, Building 345B, DK-2800 Kgs. Lyngby, Denmark; E-Mails: erik.v.thomsen@nanotech.dtu.dk (E.V.T.); ole.hansen@nanotech.dtu.dk (O.H.); 2 CINF Center for Individual Nanoparticle Functionality, Technical University of Denmark, Building 345B, DK-2800 Kgs. Lyngby, Denmark

**Keywords:** optical sensor, MEMS, Bragg grating

## Abstract

We present the design, fabrication and characterization of a new all-optical frequency modulated pressure sensor. Using the tangential strain in a circular membrane, a waveguide with an integrated nanoscale Bragg grating is strained longitudinally proportional to the applied pressure causing a shift in the Bragg wavelength. The simple and robust design combined with the small chip area of 1 × 1.8 mm^2^ makes the sensor ideally suited for remote and distributed sensing in harsh environments and where miniaturized sensors are required. The sensor is designed for high pressure applications up to 350 bar and with a sensitivity of 4.8 pm/bar (*i.e.*, 350 ×10^5^ Pa and 4.8 × 10^−5^ pm/Pa, respectively).

## 1. Introduction

All-optical sensors are characterized by their immunity to electromagnetic interference, safe operation (no risk of ignition due to short circuits) and remote sensing capabilities due to low transmission loss in optical fibers. A number of all-optical amplitude modulated sensors are known, including the Fabry–Perot [1,2] and Mach–Zehnder interferometric sensors [3]. While amplitude modulated optical sensors are capable of extremely high sensitivity, they do not lend themselves to distributed sensing since each sensor requires its own transmission line. An alternative in cases where distributed sensing is essential for system operation is frequency modulated sensors such as fiber Bragg grating (FBG) sensors [4]. Using wavelength division multiplexing large arrays of FBGs can be used for e.g., structural health monitoring and oil exploration [5]. In this paper we present the fabrication and characterization of a recently proposed nano and micro fabricated frequency modulated Bragg grating sensor for high pressure sensing in harsh environments and use in distributed and remote sensing systems [6].

## 2. Design

The basic sensing principle is based on the deformation of a waveguide with an integrated Bragg grating causing a shift Δλ*_B_* in the Bragg wavelength λ*_B_* = 2*n_e_*Λ, where *n_e_* is the effective index and Λ the grating period. If the effect of temperature, which can easily be compensated for as will be discussed later, and photoelastic effects are ignored, the Bragg wavelength shift is given by

(1) $$\Delta \lambda_{B}=2n_{e}\Delta \Lambda$$

where ΔΛ = *ε_ℓ_*Λ is the shift in grating period due to a longitudinal strain *ε_ℓ_*; it follows that the Bragg wavelength shift can be written

(2) $$\Delta \lambda_{B}=\lambda_{B}{ɛ}_{\ell }$$

**Figure 1.**
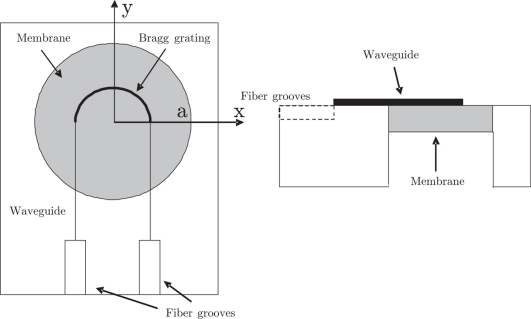
Top view (left) and side view (right) of the Bragg grating sensor. The sensor consists of a silicon membrane in a silicon chip with a dielectric optical waveguide on top; in the optical waveguide a Bragg grating is formed. The grating is located in the curved semicircular part of the waveguide on top of the membrane. The chip includes fiber alignment groves to facilitate coupling to optical fibers.

A very simple way to convert an applied pressure to a strain in the Bragg grating is to place the grating on a circular membrane. It is, however, important that the strain is uniform and longitudinal in order to increase the grating reflectivity and reduce the reflection peak bandwidth. Since the radial strain of a circular membrane is not constant, it is not practical to let the grating cross the membrane in a straight line, e.g., along a diameter. However, if the grating is placed in a waveguide following a circular curve at constant radial distance from the center of the membrane, the tangential strain at the waveguide position is constant along the length of the curve and linearly dependent on pressure and can thus be used for pressure sensing. An illustration of the device is shown in Figure 1. The effective index modulation necessary for Bragg reflection to occur can be obtained by modulating the waveguide height and/or width or by modulation of material properties. From a fabrication point of view, however, high corrugation depth combined with nanometer scale grating period and micrometer scale waveguide dimensions presents a challenge during definition of the sensor structure since high aspect ratios will occur. To avoid this problem the corrugations for this sensor are fabricated in both sides of the waveguide. Since the effective index of the waveguide is larger at the outer rim than at the inner rim, this design will effectively chirp the grating.

For isotropic plate materials the plate deflection *w* can be calculated from the well known Euler–Bernoulli equation

(3) $$D\nabla^{4}w=p$$

where *D* is the flexural rigidity and *p* is the uniform applied pressure difference across the plate. The boundary conditions for the Euler–Bernoulli equation, if the rim of the plate is rigidly clamped, are zero deflection and slope on the boundary. However, if silicon or other anisotropic materials are used as plate material, the plate deflection is better described using the Holgate–Huber equation [7]

(4) $$D_{H}\left(\frac{\partial^{4}w}{\partial x^{4}}+k_{1}\frac{\partial^{4}w}{\partial x^{3}\partial y}+k_{2}\frac{\partial^{4}w}{\partial x^{2}\partial y^{2}}+k_{3}\frac{\partial^{4}w}{\partial x\partial y^{3}}+k_{4}\frac{\partial^{4}w}{\partial y^{4}}\right)=p$$

where *k*_1_, *k*_2_, *k*_3_ and *k*_4_ are constants related to the stiffness tensor and *D_H_* the proper flexural rigidity of the anisotropic plate; for isotropic materials *k*_4_ = 1 and *k*_2_ = 2, while *k*_1_ = *k*_3_ = 0, and then Equations (3) and (4) are identical. For a crystalline silicon (100) plate *k*_4_ = 1 and *k*_2_ ≃ 2.8, while *k*_1_ = *k*_3_ = 0.

For a circular anisotropic, crystalline silicon plate of radius *a* Equation (4) is easily solved, and interestingly, the deflection shape for this specific case is identical to that of a circular isotropic plate

(5) $$w=\frac{{pa}^{4}}{64D_{\text{eff}}}{\left(1-\frac{x^{2}+y^{2}}{a^{2}}\right)}^{2}$$

however, an effective flexural rigidity *D*_eff_ = (*k*_2_ + 3*k*_4_ + 3)*D_H_*/8 must be used instead of the flexural rigidity *D*. For a (100) silicon plate *D*_eff_*/D_H_* equals (*k*_2_ + 3*k*_4_ + 3)*/*8 ≈ 1.1, while the effective flexural rigidity is

(6) $$D_{\text{eff}}=\frac{h^{3}}{12}\left[\left(C_{11}-\frac{C_{12}^{2}}{C_{11}}\right)-\frac{1}{4}\left(C_{11}-C_{12}-2C_{44}\right)\right]≃\frac{h^{3}}{12}\times 156\times 10^{9}\text{Pa}$$

where *C_ij_* are elements of the stiffness matrix for silicon. We note that the conventional treatment of the silicon (100) plate as an isotropic plate may lead to rather large errors in the calculated deflection unless elastic parameters fulfilling *Y/*(1 − *v*^2^) ≃ 156 × 10^9^ Pa are used. Hence, with the proper elastic parameters the tangential strain, *ε_θθ_*, is equal to that of a simple isotropic plate, *i.e.*, [8]

(7) $${ɛ}_{\theta \theta }=\left[1+5.72{\left(\frac{h}{a}\right)}^{2}\right]\frac{3}{8Y}\frac{\left(r^{2}-a^{2}\right)\left(1-\nu^{2}\right)}{h^{2}}p$$

where $r=\sqrt{x^{2}+y^{2}}$ and the term 5.72(*h/a*)^2^ is a correction necessary for thick plates. Combining Equations (2) and (7) the sensitivity is

(8) $$\frac{\Delta \lambda }{p}=\left[1+5.72\;{\left(\frac{h}{a}\right)}^{2}\right]\;\frac{3(r^{2}-a^{2})}{8\left[\left(C_{11}-\frac{C_{12}^{2}}{C_{11}}\right)-\frac{1}{4}(C_{11}-C_{12}-2C_{44})\right]\;h^{2}}\lambda_{B}$$

which using Equation (6) reduces to

(9) $$\frac{\Delta \lambda }{p}=\frac{1}{156\times 10^{9}\text{Pa}}\left[1+5.72{\left(\frac{h}{a}\right)}^{2}\right]\frac{3\left(r^{2}-a^{2}\right)}{8h^{2}}\lambda_{B}$$

In fabricated devices thin dielectric films are deposited on the membrane, these will increase the effective flexural rigidity of the membrane due to the added thickness and due to a possible built-in stress. A built-in stress leads to additional terms $-h_{f}\frac{\partial }{\partial x_{\beta }}\left(\sigma_{\alpha \beta }\frac{\partial w}{\partial x_{\alpha }}\right)$ on the left-hand side of Equation (4), where σ*_αβ_* is the in-plane built-in stress, *h*_f_ is the thickness of the thin film, while *x_α_* and *x_β_* are the in-plane coordinates, *i.e.*, *x*_1_ = *x*, and *x*_2_ = *y* [9]; in the case of an isotropic built-in stress σ_0_ the additional term is simply −*h*_f_*σ*_0_∇^2^*w*. From a simple minimum energy calculation using the unperturbed deflection shape, Equation (5), the effect of built-in stress may be corrected for by using a revised effective flexural rigidity *D*_eff_ = [3 + *k*_2_ + 3*k*_4_ + *h*_f_σ_0_*a*^2^*/* (2*D_H_*)] *D_H_/*8.

It is further noted that the sensor allows for an additional Bragg grating outside the plate, so that any wavelength shift not induced by pressure, e.g., temperature, may be compensated for by performing a differential measurement.

The design was also analyzed using the commercial finite element modeling (FEM) software Comsol. Taking advantage of the radial symmetry of the device, the FEM analysis can be reduced to a two dimensional anisotropic continuum analysis. Each layer was defined according to dimensions obtained during fabrication and using the materials library available in Comsol. Any measured intrinsic stress was also included in the model. Fixed boundary conditions are applied on the outer perimeter and a uniform pressure load on the upper surface. Using a triangular mesh of 25600 elements a convergent solution was found.

## 3. Fabrication

**Figure 2.**
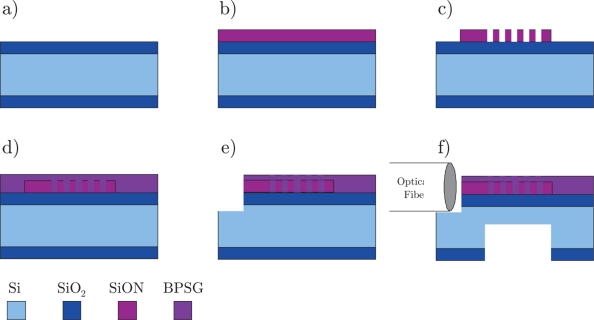
Schematic of the fabrication process. The waveguide is made on an APOX wafer, where the SiO_2_ will act as the lower cladding layer **(a)**. The waveguide core is made in SION deposited by PECVD **(b)** and the Bragg grating is fabricated by a combination of EBL and DRIE **(c)**. The upper cladding layer is made by PECVD deposited BPSG which is annealed at high temperature **(d)**. Fiber grooves **(e)** and the membrane **(f)** are both etched using AOE and DRIE.

The sensor has been fabricated according to the process flow shown in Figure 2. An atmospheric pressure oxide (APOX) silicon wafer with approximately 8 *μ*m of SiO_2_ on both sides is etched for 5 min in bHF (buffered hydrofluoric acid) in order to reduce surface roughness and remove any particles (a). The remaining SiO_2_ is acting as the lower cladding layer of the waveguide. The core of the waveguide is made from 2.5 *μ*m SiON deposited by PECVD (b). The index contrast is approximately 0.02 and the core thickness allows for single mode waveguide operation at 1,550 nm wavelength. The Bragg grating is formed in the SiON by e-beam lithography (EBL) using the positive resist ZEP520a and a lift-off of 60 nm aluminum that is used as an etch mask in the following deep reactive ion etch (DRIE) (c). The upper cladding is made of borophosphosilicate glass (BPSG) that allows for re-flow during the following 1,000 °C anneal and thereby improved step coverage of the Bragg grating; the step coverage is in general otherwise insufficient in PECVD processes (d). The fiber grooves (e) and the membrane are both made by conventional UV lithography followed by an advanced oxide etch (AOE) and a DRIE silicon etch. The resulting membranes are 135 *μ*m thick of which 112 *μ*m is silicon and 23 *μ*m is cladding layers (SiO_2_, SiON and BPSG).

**Figure 3.**
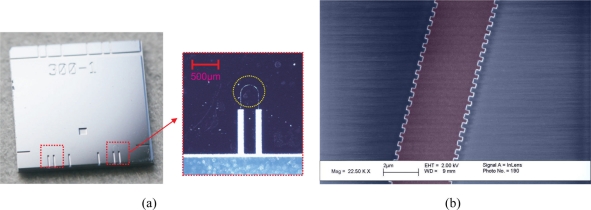
**(a)** Microscope images of a test chip with two sensors (marked with red dashed boxes). The test chip area is 1 × 1 cm^2^ while the sensors occupy an area of 1 × 1.8 mm^2^. The membrane is not visible, but has been marked with a yellow circle on the close up of the sensor. **(b)** Color enhanced SEM image of part of the curved waveguide (purple). The Bragg grating is seen as corrugations in both sides of the waveguide. The period of the Bragg grating is 520 nm.

The final test chip is shown in Figure 3(a). The test chip is relatively large, 1 × 1 cm^2^, in order to facilitate and ease handling during characterization and contains two sensors as well as additional fiber grooves. The sensors each takes up an area of 1 × 1.8 mm^2^ and have membrane radii of 400 *μ*m. A SEM image of the part of the waveguide containing the grating region is shown in Figure 3(b) at step (c) in the fabrication process. The corrugations in the sides of the waveguide are clearly seen and each have a width of 260 nm (Λ = 520 nm). In order to obtain a detectable reflection peak even for short gratings, the index modulation and thus the corrugation depth has to be sufficiently large. In this case a corrugation depth of 260 nm is used.

## 4. Results

**Figure 4.**
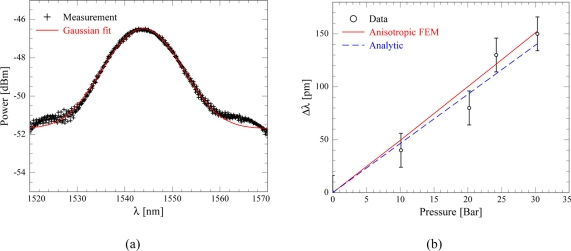
**(a)** Measured reflection spectrum from the a Bragg grating sensor with a Bragg wavelength of 1,543.8 nm; the full red curve is a Gaussian fit to the measured data with a standard deviation of the center wavelength of 16 pm. **(b)** The measured absolute Bragg wavelength shift as function of pressure for a 400 *μ*m radius membrane with the grating positioned at 200 *μ*m from the center. The error bars equal ±16 pm, *i.e.*, one standard deviation, and thus the sensitivity of ∼ 4.8 pm/bar (4.8 × 10^−5^ pm/Pa) is found to be easily within two standard deviations of both FEM simulation (full curve) and analytical results (dashed curve).

For characterization of the pressure sensitivity gas pressure was applied to the Bragg grating side of the membrane using a Druck DPI520 pressure controller and light was coupled to the test chip from a Koheras SuperK supercontinuum laser through a SMF-28 optical fiber. The reflected light was measured by means of a fiber optic circulator and an Agilent AQ-6315A optical spectrum analyzer. The spectrum of the reflected signal is seen in Figure 4(a), and from a Gaussian fit the center wavelength was found to be λ*_B_* = 1543.8 nm, while the standard deviation is 16 pm and the full width half maximum of the peak is FWHM = 17.6 nm. This relatively large peak width is a result of the chirping mentioned in the design section. The Gaussian fit is applied using the linear power scale and a power offset is included. While other fitting functions could be used, the Gaussian fit agrees well with measured data.

The change in Bragg wavelength as a function of applied pressure for a 400 *μ*m radius membrane sensor is plotted in Figure 4(b). A conservative estimate of the maximum allowable pressure, *p_max_*, is found theoretically using a silicon yield strength of ≈ 1*/*10 the actual silicon yield strength [10], yielding *p_max_* = 350 bar (350 ×10^5^ Pa). Measurement uncertainties are primarily related to the quality of the Gaussian fit and to the accuracy of the pressure read-out from the pressure controller. A linear fit of the measurement data results in a slope of 4.8 pm/bar (4.8 ×10^−5^ pm/Pa). Considering the standard deviation of the Gaussian fit is 16 pm (shown as error bars in the plot), the measurements are easily within two standard deviations of the finite element method (FEM) model (full line) and the analytical results (dashed curve) as obtained from Equation (9). The lower Young’s modulus of the cladding layers have been included in the analytical calculation by using a thickness weighted average. This reduces the effective Young’s modulus to approximately 141 MPa.

Conventional electrical MEMS pressure sensors are typically based on either piezoresistive or capacitive technology [11,12]. It is common to implement these technologies using the deflection of a membrane or plate, equivalent to the optical sensor presented here. The sensitivity of the three technologies can thus be compared by simply considering the relative change in the measured quantity, approximately given as

(10) $$\begin{array}{ccc} \frac{\Delta R}{R}=Kɛ, & \frac{\Delta \lambda_{B}}{\lambda_{B}}=ɛ, & \frac{\Delta C}{C}=\frac{w}{d_{0}} \end{array}$$

where *R* is the resistance of the piezoresistor, *K* is the piezoresistive gauge factor, *C* is the capacitance and *d*_0_ is the initial spacing and the capacitor plates. The gauge factor of p-type silicon is typically in the order of ≈50–100 [13], however, the resistor adds thermal noise and with a high resolution optical spectrum analyzer the sensitivities of the two technologies are comparable. The sensitivity of capacitive pressure sensors is typically comparable or higher than piezoresistive sensors.

The stress of the waveguide thin film was calculated from the measured change in wafer bow using Stoney’s equation [14], and an effective intrinsic stress of σ_0_ = −3.2 MPa was found; this value is so low that is does not affect the sensor sensitivity.

The coupling loss was measured to approximately 6 dB per coupling interface, which should be compared to the coupling loss of 4 dB calculated from the mode overlap integral for the fiber waveguide interface. The discrepancy may be attributed to alignment error. The transmission loss for the waveguides was measured to less than 1 dB/cm at 1,550 nm wavelength. The wavelength shift due to temperature change of the integrated Bragg grating is found by heating a test chip using a Peltier element. A temperature sensitivity of approximately 30 pm/K was found. The temperature sensitivity is primarily due to a large thermooptic coefficient of the waveguide material [15] and is comparable to the temperature sensitivities found in FBG sensors.

## 5. Conclusions

In this paper the design, fabrication and characterization of a new all-optical frequency modulated high pressure sensor has been presented. The sensor is well suited for distributed and remote sensing in harsh environments. The sensor is made with conventional MEMS materials and technology combined with e-beam lithography for nanostructuring of the Bragg grating, which allows for simple fabrication and high mechanical stability. The pressure sensitivity of the sensor was measured to be 4.8 pm/bar (4.8 × 10^−5^ pm/Pa) and the temperature sensitivity was measured to be 30 pm/K. The design could be optimized for higher or lower sensitivities by adjusting the membrane dimensions.

## References

[1] Fu H., Fu J., Qiao X. (2004). High sensitivity fiber Bragg grating pressure difference sensor. Chinese Optics Lett.

[2] Wang X., Li B., Xiao Z., Lee S.H., Roman H., Russo O.L., Chin K.K., Farmer K.R. (2005). An ultra-sensitive optical MEMS sensor for partial discharge detection. J. Micromech. Microeng.

[3] Wagner D., Frankenberger J., Deimel P.P. (1994). Optical pressure sensor using two Mach-Zehnder interferometers for the TE and TM polarization. J. Micromech. Microeng.

[4] Liu L., Zhang H., Zhao Q., Liu Y., Li F. (2007). Temperature-independent FBG pressure sensor with high sensitivity. Opt. Fiber Techn.

[5] Graham-Rowe D. (2007). Sensors take the strain. Nature Photon.

[6] Fragiacomo G., Reck K., Lorenzen L., Thomsen E.V. (2010). Novel designs for application specific MEMS pressure sensors. Sensors.

[7] Holgate C. (1946). The transverse flexure of perforated aeolotropic plates. Proc. Roy. Soc. A Mathe. Phys. Eng. Sci.

[8] Boresi A.P, Sidebottom O.M. (1985). Advanced Mechanics of Materials.

[9] Landau L.D., Lifshitz E.M. (1986). Theory of Elasticity.

[10] Petersen K.E. (1982). Silicon as a mechanical material. Proc. IEEE.

[11] Lin L., Yun W. (1998). Design, optimization and fabrication of surface micromachined pressure sensors. Mechatronics.

[12] Pedersen T., Fragiacomo G., Hansen O., Thomsen E.V. (2009). Highly sensitive micromachined capacitive pressure sensor with reduced hysteresis and low parasitic capacitance. Sens. Actuat. A Phys.

[13] Barlian A.A., Park W.-T., Mallon J.R., Rastegar A.J., Pruitt B.L. (2009). Review: Semiconductor piezoresistance for microsystems. Proc. IEEE.

[14] Stoney G.G. (1909). The tension of metallic films deposited by electrolysis. Proc. Roy. Soc. Lond.

[15] Eldada L. (2002). Polymer integrated optics: Promise *vs*. practicality. Organic Photon. Mater. Dev. IV.

